# Epidemic features and potential impact of vaccine campaigns on the 2025 chikungunya outbreak in southern China: a mathematical modelling study

**DOI:** 10.1016/j.nmni.2026.101791

**Published:** 2026-06-11

**Authors:** Qinyue Zheng, Yibo Ding, Xin Wang, Yang Yang, Qingchun Meng, Jaffar A. Al-Tawfiq, Zifu Zhong, Wenshi Wang, Qiuwei Pan

**Affiliations:** a School of International Affairs and Public Administration, Ocean University of China, Qingdao, China; b Department of Pathogen Biology and Immunology, Jiangsu Key Laboratory of Immunity and Metabolism, Jiangsu International Laboratory of Immunity and Metabolism, Xuzhou Medical University, Xuzhou, 221004, China; c Department of Gastroenterology and Hepatology, Erasmus MC-University Medical Center, Rotterdam, the Netherlands; d Shenzhen Third People's Hospital, Second Hospital Affiliated with the School of Medicine, Southern University of Science and Technology, Shenzhen, Guangdong Province, China; e National Clinical Research Center for Infectious Disease, Shenzhen, Guangdong Province, China; f School of Management, Shandong Key Laboratory of Social Supernetwork Computation and Decision Simulation, Shandong University, Jinan, China; g Infectious Disease Unit, Specialty Internal Medicine, Johns Hopkins Aramco Healthcare, Dhahran, Saudi Arabia; h Division of Infectious Diseases, Indiana University School of Medicine, Indianapolis, IN, USA; i Division of Infectious Diseases, Johns Hopkins University, Baltimore, MD, USA; j Accreditation and Infection Control Division, Quality and Patient Safety Department, Johns Hopkins Aramco Healthcare, Dhahran, Saudi Arabia; k Department of Pharmaceutics, Ghent University, Gent, Belgium

## Abstract

Chikungunya virus (CHIKV), an emerging arbovirus, is transmitted by *Aedes* mosquitoes. Climate change and increasing population mobility have driven recent outbreaks beyond traditional endemic regions. Since early July 2025, Guangdong province in southern China has faced an unprecedented outbreak of chikungunya fever. We aim to in-depth describe the epidemic features and theoretically assess the potential impact of vaccination campaigns. In total, the outbreak reported more than 25,000 cases. Foshan and Jiangmen successively emerged as epicenters of the outbreak. Through stringent public health interventions, the outbreak was controlled within two months in these two epicenters, respectively. Phylogenetic analysis revealed close genetic relation of the CHIKV isolates from this outbreak to the recent isolates in Réunion and Mayotte, but distant from the ones previously identified in China. Pre-emptive vaccination, achieving 20, 40%, 60%, and 80% coverage pre-outbreak, would avert up to 57%, 81%, 92%, and 97% infections shown by mathematical modelling, respectively. Achieving a daily vaccination rate comparable to the COVID-19 rollout, covering approximately 4% of the population in Foshan and 2% of the population in Jiangmen per day, could lower cumulative CHIKV infections by 68.7% in Foshan within two months and by 98.4% in Jiangmen within four months, respectively. In summary, the 2025 chikungunya outbreak in Guangdong was likely sparked by case importation. Implementation of stringent public health interventions is possible to control the outbreak, but can provoke significant public concerns. Vaccine campaigns are expected to be effective in both preparedness and response to future chikungunya outbreaks.

## Highlights

• Guangdong province in southern China faced two epidemic waves of chikungunya outbreak in 2025.
• Phylogenetic analysis suggests the outbreak associated with case importation.
• Stringent public health interventions is possible of controlling the outbreak, but provoke public concerns.
• Vaccine campaigns are estimated to be effective in both preparedness and response to future chikungunya outbreaks.

## 1 Introduction

Chikungunya virus (CHIKV), an emerging arbovirus, is a positive-sense RNA virus, transmitted to humans through bites of infected *Aedes* mosquitoes. CHIKV infection in humans can cause a range of clinical manifestations including fever, headache and rash, and an estimated 0.3% of patients with chikungunya die [1]. Many patients develop prolonged, debilitating joint pain lasting months to years, severely affecting quality of life and productivity [2]. Climate change and the increasing frequency of extreme weather events have created favorable conditions for mosquito proliferation and sustained arbovirus transmission. In recent years, chikungunya cases have been reported in more than 100 countries, reflecting a marked expansion beyond traditional endemic regions [3,4]. In 2025, a notable surge has occurred, with outbreaks emerging in temperate regions such as Europe [5] and parts of Asia [6], historically considered non-endemic for CHIKV.

With no licensed antivirals available, supportive care remains the mainstay of treatment but is often inadequate to prevent chronic sequelae. These challenges highlight the urgent need for effective preventive measures. To date, two chikungunya vaccines have recently been licensed: a live-attenuated vaccine (IXCHIQ) and a virus-like particle vaccine (VIMKUNYA). Their regulatory approval by the US Food and Drug Administration (FDA), European Medicines Agency (EMA), and UK's Medicines and Healthcare products Regulatory Agency (MHRA) represents a pivotal milestone.

Since early July 2025, an unprecedented chikungunya outbreak has emerged in Guangdong province, southern China. This study aims to comprehensively describe the epidemic features and to assess the potential impact of vaccination campaigns on the current outbreak by mathematical modeling.

## 2 Methods

### 2.1 Model settings

The 2025 chikungunya outbreak in Guangdong province, China was modeled mainly based on a previous study assessing the potential benefits of a vaccination campaign [7]. The system of differential equations was described as followed:

(1) $$\frac{dS}{dt}=-\beta \left(t\right)*\frac{I+IV}{N}*S-va*S$$

(2) $$\frac{dV}{dt}=-\beta \left(t\right)*\frac{I+IV}{N}*\left(1-ve\right)*V+va*S$$

(3) $$\frac{I}{dt}=\beta \left(t\right)*\frac{I+IV}{N}*S-\gamma *I$$

(4) $$\frac{IV}{dt}=\beta \left(t\right)*\frac{I+IV}{N}*\left(1-ve\right)*V-\gamma *IV$$

(5) $$\frac{dR}{dt}=\gamma *I$$

(6) $$\frac{dRV}{dt}=\gamma *IV$$

The total population (N) as grouped as six subpopulations: susceptible and unvaccinated (S), susceptible and vaccinated (V), infected and unvaccinated (I); infected and vaccinated (IV); recovered and unvaccinated (R); recovered and vaccinated (RV).

β(t) is the real-time transmission rate, which is calculated by time-varying reproduction number $R_{t}$ and recovery rate γ. We adopted frequency-dependent transmission to reflect macroscopic population dynamics, where the effective parameter β(t) integrates the impacts of local Aedes clustering, human mobility and spatial heterogeneity.

### 2.2 Scenario settings

We modeled two distinct vaccination strategies using a dynamic transmission framework calibrated to Guangdong's outbreak dynamics. Scenario 1 (preparedness): Vaccination coverage was achieved pre-outbreak across susceptible populations at 40%, 69%, 78%, 90% in Foshan City and 30%, 60%, 90% in Jiangmen City. Given China's 90% COVID-19 vaccination coverage, an estimated 10% of the population are persistent vaccine refusers. Among the maximum 90% of the population that are generally vaccine-accepting, for VIMKUNYA, which is approved by the U.S. FDA for individuals aged 12 years and older, the maximum achievable vaccination coverage is 78% of the total population; for IXCHIQ, which is explicitly contraindicated for adults aged 65 years and older by UK public health authorities, the maximum eligible coverage is further reduced to 69% after excluding the older age group prohibited from vaccination. We follow the base case setting adopted in previous published studies and specify a baseline vaccine coverage of 40% in our main analysis.

Scenario 2 (outbreak response): Vaccination commenced after epidemic onset, with daily administration rates of 0.1%-1.0% of the population in Foshan City and 1.0–3.0% of the population in Jiangmen City sustained until the pandemic being significantly suppressed. Critically, a rate of 2.3% per day replicates Guangdong ’s peak COVID-19 rollout capacity [8]. While, the capacity is limited when compare to COVID-19 vaccination, and that actual rollout speed for a chikungunya vaccine would likely be slower due to the absence of emergency authorization and existing infrastructure. For comparability, the same parameterization as in previous studies was adopted with a 40% vaccine coverage over a three-month period, which corresponds to a daily vaccination rate of approximately 0.4% [9].

### 2.3 Time-varying reproduction number

$R_{t}$ represents the near-real-time number of secondary cases generated by per infected individual. It was estimated based on the real-world daily incidences data using R package EpiEstim [10]. This method has been widely used in epidemic studies [11,12]. The mean serial interval and its standard deviation were calculated using values of the shape and rate parameters for the serial interval distribution of 14.69 and 0.64, respectively, observed by pervious work [13]. Accordingly, the serial interval (SI) and its standard deviation were calculated as 22.95 days and 5.99. Similar values of SI were reported in the literature, at 1.5 to 2.7 weeks [14] and 23 days [15], respectively. Given the wide range of the serial interval, the estimated time-varying reproduction number and its 95% credible interval were highly volatile in the initial phase; therefore, estimation was initiated from three weeks after the first case.

### 2.4 Initial values, key parameters, and data source

The values were initialized according to the real-world settings. The total population N is 9.69 million in Foshan and 4.82 million in Jiangmen. $V_{0}=N*va$, $S_{0}=N*\left(1-va\right)-I_{0}$, $I_{0}=1$, ${IV}_{0}=$ 0, $R_{0}=0$, ${RV}_{0}=0$.

The recovery rate γ=1/σ, where σ is the mean duration of infectiousness, fixed at 15 days. va denotes the cumulative coverage of vaccine against chikungunya virus (CHIKV). The efficacy of vaccines in protecting individuals from infections is ve. We mainly employed parameter estimates from a recent study that analyzed an epidemic in Paraguay as a base case study, assuming a vaccine efficacy of 40% against infection and 70% against disease [9]. For sensitivity analysis, ranges of 40–70% and 70–95% are set for infection protection and disease protection, respectively, in the Jiangmen scenario [16]. The vaccine-induced protection against symptomatic infections is calculated by comparing the chance of being symptomatic between vaccinated ($\delta_{v}$) and unvaccinated ($\delta_{u}$) infections, using real-world reported case counts. The parameters were sourced from widely cited studies on CHIKV. The values of parameters are listed in Table S1.

Original epidemic data were obtained from the Guangdong Provincial Center for Disease Control and Prevention (CDC) (https://cdcp.gd.gov.cn/ywdt/zdzt/yfjkkyr/, accessed December 08, 2025).

### 2.5 Phylogenetic analysis

Full-length CHIKV sequences from China (n = 116) were retrieved from GenBank (https://www.ncbi.nlm.nih.gov/genbank) on December 10, 2025. Additionally, full-length CHIKV sequences released after 2024 were collected using predefined search keywords: chikungunya [All Fields] AND “Alphavirus chikungunya” [porgn] AND ((“10000" [SLEN]: “20000" [SLEN]) AND (“2024/01/01" [PDAT]: “2025/12/30" [PDAT])). After removal of duplicate sequences, 536 full-length sequences were retained, with associated metadata (accession numbers, collection dates, geographic locations). An initial maximum-likelihood phylogenetic tree (ML tree) was constructed using FastTree (GTR + G4, 1000 ultrafast bootstraps) [17] to identify subclades and assess sequence redundancy. Subclades were defined based on monophyletic groups with bootstrap support ≥70%. Based on the annotated subclade information and assisted by SeqKit, we performed standardized and fixed-seed random subsampling via a custom PowerShell script (random seed = 1234), which has been uploaded as supplementary material. The subsampling criteria were strictly followed: (1) all sequences originating from China were completely retained to preserve domestic genetic diversity; (2) non-Chinese sequences were grouped by annotated subclades and geographic locations, with one representative sequence randomly selected per group to balance phylogenetic topology and geographic representativeness. After subsampling, a final dataset of 210 sequences was generated for formal phylogenetic reconstruction.

The final maximum-likelihood phylogenetic tree was reconstructed using IQ-TREE (version 2.2.0) [18]. The nucleotide substitution model TIM2+F + I + G4 was selected based on the Bayesian Information Criterion (BIC) implemented in ModelFinder. Branch reliability was evaluated using 1000 ultrafast bootstrap (UFBoot) replicates and the SH-like approximate likelihood ratio test (SH-aLRT). Statistically robust branch support was defined as SH-aLRT ≥80% and UFBoot ≥90%. Genotype assignment was performed according to a previous study [19]. The O'nyong'nyong virus (GenBank: NC_001512.1) was used as the outgroup to root the tree. Detailed information on the sequences and methods of the analysis were included as an additional supplementary information.

## 3 Results

**Fig. 1:**
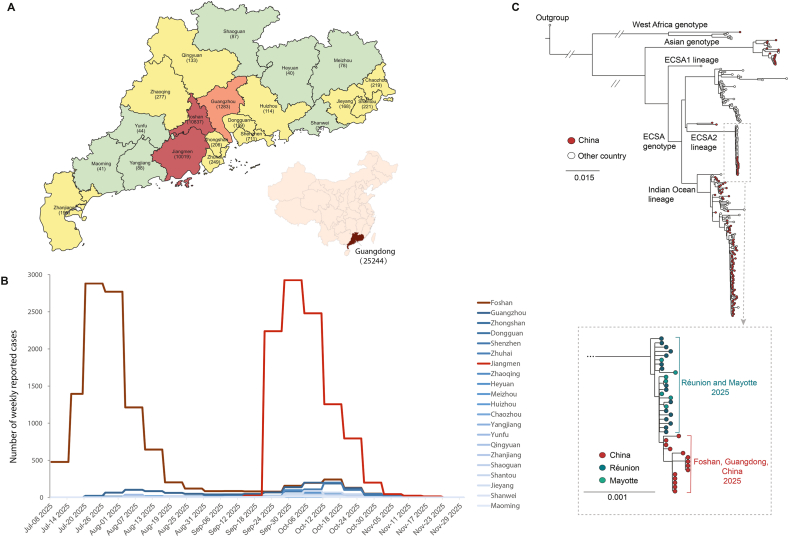
**Epidemic dynamics of the 2025 chikungunya outbreak in southern China and phylogenetic features of the viral strain. A.** Map illustrating epidemic burden across different cities of Guangdong province, with warmer colors marking higher cumulative infection counts as of mid-November, 2025. A bar chart summarizes weekly incidence in each city. The total reported cases for Guangdong and the neighboring province Guangxi were indicated. **B.** A line chart summarizes weekly incidence in each city of Guangdong province, China. **C.** Maximum-likelihood phylogenetic tree of full-length Chikungunya virus genomes. Major genotypes and lineages are indicated. Geographic regions are colour-coded as shown. A subclade within the East/central/south African clade 2 (ECSA2) lineage is enlarged to show clustering of isolates from the Foshan outbreak, Guangdong, China (red), with recent isolates from Réunion (dark green) and Mayotte (green). Scale bars indicate nucleotide substitutions per site. The O'nyong'nyong virus (GenBank: NC_001512.1) was used as the outgroup to root the tree.

Since early July 2025, a major chikungunya outbreak has emerged in Guangdong province, southern China, with over 25,000 cases reported in total (Fig. 1A). The outbreak was triggered by an imported case to Foshan, a major industrial hub of the province. By the time the first case was identified on July 8, undetected ongoing transmission was likely already occurring, as daily reported cases exceeded 200 by July 13. As the first epicenter, Foshan recorded 92.6% (9703) of the total cases in Guangdong province in July and August (Fig. 1B). Following containment of the outbreak in Foshan, a neighboring city, Jiangmen, reported an unexpected surge in cases. Despite only 14 cumulative cases during July and August, weekly incidence exceeded 2000 in the third week of September, culminating in cumulative cases surpassing 10,000 by November 15 (Fig. 1B), which emerged as an epicenter of the second epidemic wave. The virus rapidly spread to other areas, notably Guangzhou (1283 reported cumulative cases) and Shenzhen (711 reported cumulative cases), two megacities with a population of more than 20 million. It has also extended to neighboring Guangxi province, and to adjacent regions including Hong Kong and Macau, likely driven by population mobility.

Phylogenetic analysis showed that sequenced CHIKV strains in the current outbreak clustered within the East/Central/South African clade 2 (ECSA2) lineage (Fig. 1C). These isolates were closely related to recent strains from Réunion and Mayotte but genetically distinct from previously reported CHIKV strains in China (Fig. 1C), indicating a recent external introduction rather than persistence of earlier local lineages.

**Fig. 2:**
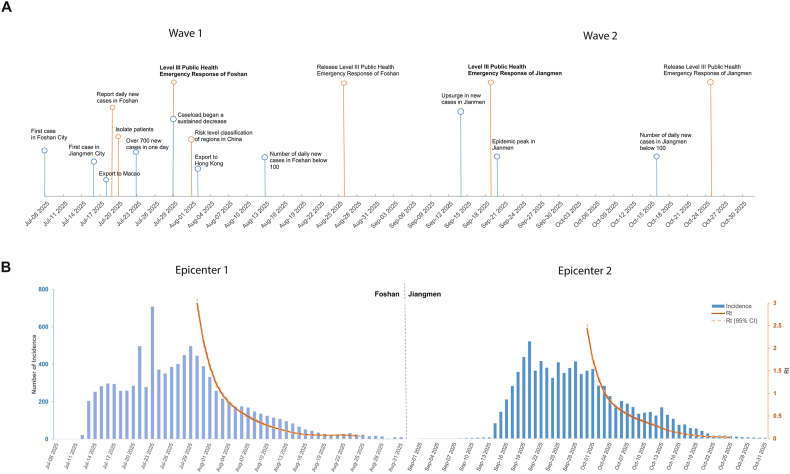
**Epidemic features and public health responses during the 2025 chikungunya outbreak in two epicenters. A.** Timeline of key outbreak events, with blue lines indicating critical epidemic and transmission dates, and orange lines denoting major public health intervention dates. **B.** The estimated real-time reproduction number ($R_{t}$) and daily number of new cases reported in Epicenter 1 (Foshan) and subsequently Epicenter 2 (Jiangmen) of the outbreak. The solid line represents the mean value of $R_{t}$, and the dashed line denotes its 95% credible interval.

To combat this outbreak, a series of public health measures were swiftly implemented, including the activation of a Level III public health emergency response in Foshan on July 29. China's public health emergency response system is divided into four tiers based on the scope and severity of the incident, corresponding to: Level 1 (Extraordinarily Serious), Level 2 (Major), Level 3 (Larger-scale), and Level 4 (Ordinary). The decision to activate a Level 3 Public Health Emergency Response is made by the people's government of a prefecture-level city or above. A series of targeted public health measures were implemented, encompassing intensive disease awareness campaigns penetrating deep into communities, while simultaneously establishing a robust diagnostic network by designating 40 hospitals capable of performing specific nucleic acid testing for the Chikungunya virus [20]. Alongside mobilized efforts encouraging both residents and factories to eliminate mosquito breeding grounds through the removal of stagnant water and the application of insecticides, including deployment of drones for mosquito control, release of sterilized male mosquitoes, isolation of infected individuals, the release of *Toxorhynchites splendens* as a natural predator to suppress *Aedes albopictus* populations (Fig. 2A). Together, these interventions led to a sustained decline in daily new cases from early August, with the reproductive number decreasing to well below 1 by the end of the month. Therefore, the Level III public health emergency was terminated on August 26, and the outbreak was finally under control within two months. In response to the unexpected surge of the second wave in Jiangmen, a Level III public health emergency response was again activated on September 10 (Fig. 2A). Within two weeks, the reproductive number fell below 1 in Jiangmen (Fig. 2B), leading to termination of the emergency declaration on October 25 (Fig. 2A).

**Fig. 3:**
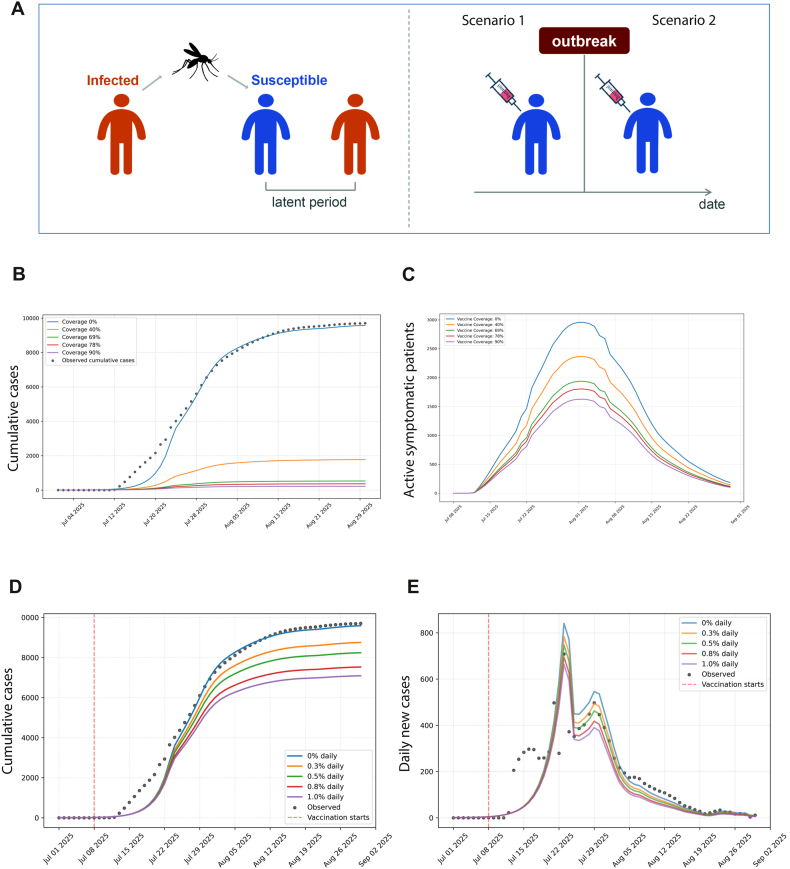
**Simulating the impact of vaccine campaigns with two scenarios on the 2025 chikungunya outbreak in Foshan. A**. CHIKV is transmitted by mosquito bites, leading to clinical symptoms after an incubation period. Simulation models assess vaccination campaigns launched before the outbreak (scenario 1; panels B and C) and in response to the outbreak (scenario 2; panels D and E), evaluating their effects on epidemic burden. Cumulative cases (**B**) and cumulative symptomatic infections (**C**) under varying pre-outbreak vaccination coverage levels (0%, 40%, 69%, 78% and 90%). The grey dots denote the observed cumulative cases during this outbreak. Cumulative cases (**D**) and daily new cases (**E**) projected for different daily vaccination rates (0%, 0.3%, 0.5%,0.8% and 1.0%) when vaccination begins after the outbreak starts in Foshan (July 8). The dash line indicates the start date of the vaccine campaign. The grey dots show the observed cumulative cases and daily new cases throughout this outbreak.

**Fig. 4:**
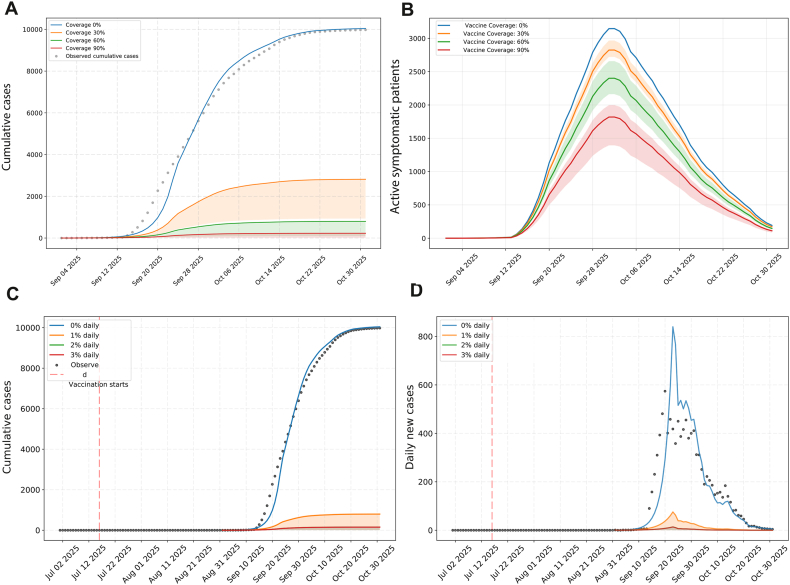
**Simulating the impact of vaccine campaigns on the 2025 chikungunya outbreak in Jiangmen.** In scenario 1, cumulative cases (**A**) and cumulative symptomatic infections (**B**) across varying coverage levels of pre-outbreak vaccination under varying pre-outbreak vaccination coverage levels (0%, 30%, 60% and 90%). In scenario 2, cumulative cases (**C**) and daily new cases (**D**) projected for different daily vaccination rates (1.0%, 2.0% and 3.0%), with vaccination initiated following the onset of the Jiangmen outbreak (July 16).

Given the absence of a licensed chikungunya vaccine in China, we performed mathematical modeling to assess the potential impact of vaccination campaigns on the current outbreak, focusing on the two epicenters, Foshan and Jiangmen. We first simulated a preparedness scenario in which vaccines were available prior to the outbreak. Vaccinating susceptible individuals at coverage levels of 40%, 69%, 78%, and 90% in Foshan was estimated to reduce cumulative infections by 81.42%, 94.45%, 96.17%, and 97.66%in two months, respectively (Fig. 3B; Table S2). Correspondingly, this would prevent 20%, 35%, 39%, and 45% of individuals from developing debilitating symptoms (Fig. 3C; Table S3). Simulation in Jiangmen also demonstrates the critical impact of vaccination timing and coverage on outbreak trajectory. In the preparedness scenario, when considering ‌sensitivity‌ analysis of vaccine efficacy, pre-outbreak vaccination in Jiangmen reduced cumulative symptomatic cases by 71.9% (71.9%-90.7%), 92.0% (92.0%-99.1%), and 97.7% (97.7%-99.9%) at vaccine coverage rates of 30%, 60%, and 90%, respectively, within two months (Fig. 4A; Table S4). Concurrently, asymptomatic infections decreased by 89.8% (86.5%-94.3%), 76.3% (68.7%-84.5%), and 57.8% (44.3%-63.5%) versus the no-vaccination baseline (Fig. 4B; Table S5).

We next modeled an outbreak response scenario, initiating vaccination after local transmission had started. In this case, administering vaccines to 0.1–1.0% of the population daily could markedly reduce transmission and disease burden (Tables S6 and S7). A daily vaccination rate of approximately 0.5% of the total population reduced cumulative infections by 14.4% within two months (Fig. 3D and E; Table S6). At a daily vaccination rate of 1%, cumulative infections were reduced by 26.1% (Table S6). Even though a surge in cases emerged in Foshan within one week of vaccine rollout, strategic planning and timely resource allocation for vaccination nonetheless remained pivotal to mitigating the epidemiological impact. While, the epidemiological trajectory in Jiangmen differed substantially: the index case in Jiangmen was documented on 16 July. Driven by a daily vaccination rate of 2%, in line with Jiangmen's maximum COVID-19 vaccine rollout capacity, vaccine coverage approached universal coverage by the time of the subsequent case surge two months later, with cumulative infections reduced by 98.5% (98.5%-99.9%) over the intervention period (Fig. 4C and D; Tables S8 and S9). Even with a more modest daily vaccination rate of 1%, vaccine coverage had exceeded 60% by the mid-September epidemic onset, affording protection against infection to 92.1% (92.1%-98.7%) of the population, demonstrating that even modest vaccination rates can suppress outbreak trajectories when deployed prior to exponential growth phases.

## 4 Discussion

Guangdong is China's most populous province, home to 127 million people. As the country's primary business and economic hub, it experiences extensive domestic and international population mobility [21]. Combined with its subtropical climate and widespread mosquito vectors, the region faces a high risk of arboviral epidemics, with potential for rapid spread both to neighboring areas and internationally. A similar situation is seen in Europe, where countries such as France and Italy face repeated importation of chikungunya cases linked to travel [22]. With warming weather and the wide distribution of key mosquito species (e.g., *Aedes albopictus*), such imported cases have triggered unprecedented local transmission [22,23]. Phylogenetic analysis in this study revealed that the strain responsible for the current outbreak in Guangdong is genetically distant from previous isolates identified in China [[24], [25], [26], [27]], yet closely related to strains recently detected in Réunion and Mayotte. In 2024 and 2025, Réunion and Mayotte experienced major chikungunya outbreaks with widespread local transmission, linked to a specific viral lineage adapted to *Aedes albopictus* mosquitoes [28]. These findings suggest that the outbreak in Guangdong was likely triggered by case importation, consistent with epidemiological evidence of an imported case reported in Foshan at the onset of the outbreak.

Guangdong's experience demonstrates that rapid and decisive action, including non-pharmaceutical interventions, can contain outbreaks relatively quickly. However, such coordinated and stringent responses are not universally feasible and may provoke public concern, recalling experiences from China's ‌strict control measures during the early COVID-19 epidemic [29]. Furthermore, these public health responses alone are not sustainable for long-term outbreak preparedness. Shortly after the first wave was contained in the Foshan epicenter, a second wave emerged in Jiangmen, although this was again controlled through stringent control measures including activation of a Level III public health emergency response.

Although no fatalities were reported during this outbreak, the impact of chikungunya extends far beyond mortality. CHIKV infections are frequently misdiagnosed or underreported due to the similarity of early symptoms with other arboviral diseases, particularly dengue fever, complicating timely diagnosis and intervention [30]. Unlike dengue, which typically resolves within weeks, chikungunya can progress to chronic arthropathy requiring long-term management and imposing substantial personal and economic burdens [4]. In light of these challenges, vaccine deployment is crucial. The rapid onset of protective immunity offered by available chikungunya vaccines makes them particularly suitable for emergency immunization campaigns during outbreaks [31]. In regions at risk, especially non-endemic areas with high inbound travel, pre-emptive vaccination of travelers and vulnerable populations could serve as a frontline defense. In endemic low-income tropical regions, widespread vaccination could mitigate long-term societal impact. In this study, we modeled two scenarios: one in which vaccines were available before the outbreak (preparedness) and another where vaccination was initiated after outbreak onset (response). Integrating vaccination into comprehensive public health strategies, both scenarios showed clear benefits, including sharply reducing incidence and overall burden, indirectly lowering long-term disability, and easing pressure on health systems. Our findings are largely in line with modelling studies for other epidemic and endemic settings such as Paraguay [9] and Brazil [7].

Of note, surveillance following deployment of the live-attenuated IXCHIQ vaccine reported serious adverse events, including hospitalizations and deaths due to chikungunya-like illness and encephalitis [32]. The US FDA suspended its license in August 2025, and the UK's Commission on Human Medicines has temporarily restricted its use in people aged over 65 [33]. Currently, the virus-like particle vaccine VIMKUNY remains available, providing protection against chikungunya for adolescents from age 12. Based on pooled data from five completed clinical studies involving 3522 participants with six months of follow-up, this vaccine demonstrated an acceptable safety profile, with the most commonly reported side effects being tiredness, headache, muscle pain, and injection-site pain [34]. Early post-authorisation data from the United States and Germany also support its favorable safety profile in ≥65-year-olds [35]. When weighing the risk of potential severe adverse events following vaccination against the typically non-lethal risk of natural infection, the prevalence of vaccine hesitancy is expected to rise, representing a major barrier to achieving high vaccination coverage.

Our study has several limitations. First, in the absence of traditional phase 3 trials, vaccine efficacy data against infection or disease are not available. The key parameter values used here, as in previous studies [4,9], were based on expert consensus from Gavi, the World Health Organization, and academia. Second, since no deaths were reported during this outbreak, we could not assess potential effects on reducing mortality. Third, we modeled vaccination only in combination with the real-world public health responses implemented during the Guangdong outbreak. Finally, while Chikungunya virus transmission is biologically dependent on Aedes vectors, the lack of temporal data on local mosquito density and our model employs a simplified transmission framework that does not explicitly include mosquito compartments. We will prioritize this direction in future work; once fine-scale vector surveillance data becomes available, we will more accurately capture the impact of temperature variation and different vector control modalities (e.g., source reduction vs. sterile male releases) on local transmission.

Despite these limitations, our study provides an in-depth characterization of the epidemic features of the 2025 chikungunya outbreak in southern China and offers strong evidence supporting the benefits of vaccine campaigns for outbreak response and preparedness. Future research should focus on optimizing the integration of vaccination strategies with varying intensities of other public health measures.

## IRB approval statement

This study used only publicly available, de-identified aggregate epidemiological data, and did not involve direct interaction with human subjects or collection of identifiable private information. No formal Institutional Review Board (IRB) approval was required.

## CRediT authorship contribution statement

**Qinyue Zheng:** Conceptualization, Data curation, Formal analysis, Funding acquisition, Investigation, Methodology, Project administration, Visualization, Writing – original draft. **Yibo Ding:** Data curation, Formal analysis, Investigation, Methodology, Visualization, Writing – review & editing. **Xin Wang:** Investigation, Validation, Writing – review & editing. **Yang Yang:** Data curation, Investigation, Validation, Writing – review & editing. **Qingchun Meng:** Investigation, Validation, Writing – review & editing. **Jaffar A. Al-Tawfiq:** Investigation, Validation, Writing – review & editing. **Zifu Zhong:** Investigation, Validation, Writing – review & editing. **Wenshi Wang:** Investigation, Supervision, Validation, Writing – review & editing. **Qiuwei Pan:** Conceptualization, Investigation, Supervision, Validation, Writing – original draft.

## Declaration of competing interest

The authors declare that they have no known competing financial interests or personal relationships that could have appeared to influence the work reported in this paper.
