# Supplementary material for: Epidemic features and potential impact of vaccine campaigns on the 2025 chikungunya outbreak in southern China: a mathematical modelling study
Source: New Microbes New Infect. 2026 Jun 11;72:101791. doi: 10.1016/j.nmni.2026.101791 (PMC13277609; doi:10.1016/j.nmni.2026.101791)
Supplement: Multimedia component 1 [file mmc1.pdf]

Supplementary Information

Table S1 | Values of key parameters

Parameter	Definition	Base case (Sensitivity)	Source
$\beta(t)$	time-varying transmission rate	estimated	(CDC, 2025)
γ	recovery rate	1/15	(Ribeiro dos Santos et al., 2025)
va	vaccine coverage	Assumed (0-90%)	
ve	efficacy of vaccine protection against infection	40% (40%-75%)	(Ribeiro dos Santos et al., 2025; OscarOscar Cortes-Azuero et al., 2026)
δ_v	chance of being symptomatic in vaccinated infections	25% (16.7%-25%)	(Ribeiro dos Santos et al., 2025; OscarOscar Cortes-Azuero et al., 2026)
δ_u	chance of developing symptoms in unvaccinated infections	50%	(Ribeiro dos Santos et al., 2025)

Table S2 | Reduction of cumulative infections benefited from vaccination prior to outbreak in Foshan.

	Vaccine coverage prior to outbreak			
	40%	69%	78%	90%
Jul 15	59.6%	78.9%	82.7%	86.7%
Jul 22	74.7%	90.6%	93.0%	95.3%
Jul 29	79.5%	93.5%	95.4%	97.1%
Aug 05	80.8%	94.1%	95.9%	97.5%
Aug 12	81.2%	94.3%	96.1%	97.6%
Aug 19	81.3%	94.4%	96.1%	97.6%
Aug 26	81.4%	94.4%	96.2%	97.7%
Aug 30	81.4%	94.4%	96.2%	97.7%

Table S3 | Reduction of active symptomatic cases benefited from vaccination prior to outbreak in Foshan.

	Vaccine coverage prior to outbreak			
	20%	40%	60%	80%
Jul 15	20%	35%	39%	45%
Jul 22	20%	35%	39%	45%
Jul 29	20%	35%	39%	45%
Aug 05	20%	35%	39%	45%
Aug 12	20%	35%	39%	45%
Aug 19	20%	35%	39%	45%
Aug 26	20%	35%	39%	45%
Aug 30	20%	35%	39%	45%

Table S4 | Reduction of cumulative infections benefited from vaccination prior to outbreak in Jiangmen.

	Vaccine coverage prior to outbreak		
	30%	60%	90%
Sep 08	28.48%-46.51%	48.68%-70.99%	63.02%-83.95%
Sep 15	49.39%-71.94%	74.20%-91.84%	86.71%-97.46%
Sep 22	64.38%-85.46%	87.20%-97.79%	95.34%-99.62%
Sep 29	69.74%-89.29%	90.76%-98.79%	97.14%-99.84%
Oct 06	71.16%-90.21%	91.60%-98.99%	97.52%-99.88%
Oct 13	71.63%-90.50%	91.87%-99.05%	97.63%-99.89%
Oct 20	71.88%-90.65%	92.00%-99.07%	97.69%-99.89%
Oct 27	71.94%-90.69%	92.04%-99.08%	97.70%-99.89%
Oct 31	71.95%-90.70%	92.04%-99.08%	97.71%-99.89%

Table S5 | Reduction of active symptomatic cases benefited from vaccination prior to outbreak in Jiangmen.

	Vaccine coverage prior to outbreak		
	30%	60%	90%
Sep 08	10.23% (5.70%-13.50%)	23.68% (15.52%-31.26%)	42.19% (36.49%-55.69%)
Sep 15	10.23% (5.70%-13.50%)	23.68% (15.52%-31.26%)	42.19% (36.49%-55.69%)
Sep 22	10.23% (5.70%-13.50%)	23.68% (15.52%-31.26%)	42.19% (36.49%-55.69%)
Sep 29	10.23% (5.70%-13.50%)	23.68% (15.52%-31.26%)	42.19% (36.49%-55.69%)
Oct 06	10.23% (5.70%-13.50%)	23.68% (15.52%-31.26%)	42.19% (36.49%-55.69%)
Oct 13	10.23% (5.70%-13.50%)	23.68% (15.52%-31.26%)	42.19% (36.49%-55.69%)
Oct 20	10.23% (5.70%-13.50%)	23.68% (15.52%-31.26%)	42.19% (36.49%-55.69%)
Oct 27	10.23% (5.70%-13.50%)	23.68% (15.52%-31.26%)	42.19% (36.49%-55.69%)
Oct 31	10.23% (5.70%-13.50%)	23.68% (15.52%-31.26%)	42.19% (36.49%-55.69%)

Table S6 | Reduction of cumulative cases benefited from vaccination campaign since outbreak in Foshan.

	Daily percent of the population receiving vaccines			
	0.3%	0.5%	0.8%	1.0%
Jul 15	1.1%	1.9%	3.0%	3.7%
Jul 22	4.6%	7.5%	11.7%	14.4%
Jul 29	7.0%	11.3%	17.5%	21.4%
Aug 05	8.0%	12.9%	19.8%	24.1%
Aug 12	8.4%	13.5%	20.7%	25.2%
Aug 19	8.6%	13.8%	21.2%	25.7%
Aug 26	8.7%	14.0%	21.4%	25.9%
Aug 30	8.7%	14.1%	21.5%	26.1%

Table S7 | Reduction of daily new cases benefited from vaccination campaign since outbreak in Foshan.

	Daily percent of the population receiving vaccines			
	0.3%	0.5%	0.8%	1.0%
Jul 15	1.8%	1.2%	1.9%	1.2%
Jul 22	6.1%	4.1%	6.1%	4.1%
Jul 29	9.6%	6.5%	9.6%	6.6%
Aug 05	11.7%	8.0%	11.8%	8.0%
Aug 12	13.2%	9.0%	13.4%	9.1%
Aug 19	14.2%	9.8%	14.9%	10.4%
Aug 26	16.0%	10.1%	16.1%	11.3%
Aug 30	16.0%	10.4%	17.4%	11.9%

Table S8 | Reduction of cumulative cases benefited from vaccination campaign since outbreak in Jiangmen.

	Daily percent of the population receiving vaccines		
	1%	2%	3%
Sep 08	42.95%-62.28%	66.27%-84.52%	66.82%-84.95%
Sep 15	70.36%-87.83%	89.14%-97.69%	89.32%-97.76%
Sep 22	85.94%-88.73%	96.60%-98.91%	96.66%-98.94%
Sep 29	90.36%-98.25%	98.02%-99.87%	98.05%-99.87%
Oct 06	91.44%-98.57%	98.31%-99.90%	98.34%-99.90%
Oct 13	91.77%-98.66%	98.38%-99.91%	98.41%-99.91%
Oct 20	91.99%-98.72%	98.43%-99.91%	98.46%-99.91%
Oct 27	92.05%-98.73%	98.45%-99.91%	98.47%-99.92%
Oct 31	92.06%-98.73%	98.45%-99.91%	98.48%-99.92%

Table S9 | Reduction of daily new cases benefited from vaccination campaign since outbreak in Jiangmen.

	Daily percent of the population receiving vaccines		
	1%	2%	3%
Sep 08	54.71%-76.15%	79.60%-95.45%	79.94%-95.58%
Sep 15	77.66%-93.20%	93.71%-99.42%	93.81%-99.44%
Sep 22	89.86%-98.33%	98.06%-99.93%	98.09%-99.93%
Sep 29	93.70%-99.30%	98.96%-99.98%	98.97%-99.98%
Oct 06	94.89%-99.53%	99.16%-99.98%	99.17%-99.98%
Oct 13	95.40%-99.62%	99.22%-99.99%	99.24%-99.99%
Oct 20	95.86%-99.71%	99.28%-99.99%	99.29%-99.99%
Oct 27	96.10%-98.60%	99.30%-99.93%	99.31%-99.93%
Oct 31	96.14%-99.19%	99.30%-99.96%	99.31%-99.96%

References

CDC, G. 2025. *Special Focus on Chikungunya* [Online]. Available: https://cdcp.gd.gov.cn/ywdt/zdzt/yfjkkyr/ [Accessed December 08 2025].

CORTES-Azuero O, O'DRISCOLL M, RIBEIRO dos Santos G et al. The epidemiology of chikungunya virus in Brazil and the potential impact of vaccines: a mathematical modelling study. *The Lancet Infectious Diseases*, 2025; 26, 406-416

PERKINS, T. A., METCALF, C. J., GRENFELL, B. T. & TATEM, A. J. (2015). Estimating drivers of autochthonous transmission of chikungunya virus in its invasion of the americas. *PLoS Curr,* 7.

RIBEIRO DOS SANTOS, G., JAWED, F., MUKANDAVIRE, C., DEOL, A., SCARPONI, D., MBOERA, L. E. G., SERUYANGE, E., POIRIER, M. J. P., BOSOMPRAH, S., UDEZE, A. O., DELLAGI, K., HOZé, N., CHILONGOLA, J., NASRALLAH, G. K., CAUCHEMEZ, S. & SALJE, H. (2025). Global burden of chikungunya virus infections and the potential benefit of vaccination campaigns. *Nature Medicine,* 31**,** 2342-2349.
